# Chronological age is differentially associated with cognitive performance according to climacteric stage: evidence from a Bayesian multivariate analysis in Chilean women

**DOI:** 10.3389/fpsyg.2026.1823236

**Published:** 2026-05-29

**Authors:** Jonathan Lühr-Henríquez, Matías Castillo-Aguilar, Rosa Jurado-Barba, Cinthya Velásquez Muñoz, Cristian Núñez-Espinosa

**Affiliations:** 1 Escuela de Doctorado, Facultad HM de Ciencias de la Salud, Universidad José Camilo Cela, Madrid, Spain; 2 Departamento de Psicología, Universidad de Magallanes, Punta Arenas, Chile; 3 Escuela de Medicina, Universidad de Magallanes, Punta Arenas, Chile; 4 Faculty of Health Sciences - HM Hospitals, Camilo Jose Cela University, Madrid, Spain; 5 HM Hospitals Health Research Institute, Madrid, Spain

**Keywords:** ACE-R, age, Bayesian analysis, climacteric stage, cognition, menopause, SDMT

## Abstract

**Background:**

Women traversing menopause experience neuropsychological changes affecting cognitive domains such as memory, attention, and processing speed. However, the relationship between chronological age and cognitive performance across different climacteric stages remains poorly understood, particularly in Latin American populations. The menopausal transition is increasingly recognized as a critical period for women’s cognitive health, characterized by profound neuroendocrine changes that may influence trajectories of cognitive aging. Chronological age was associated with better cognitive performance in early menopause, whereas in advanced postmenopause it was associated with lower performance. This pattern may reflect stage-specific differences related to reproductive aging, particularly across different phases of the climacteric.

**Objectives:**

To examine whether chronological age differentially modulates cognitive performance according to climacteric stage in Chilean women from the Magallanes Region.

**Methods:**

Three hundred sixty women aged 50–81 years were recruited and classified by climacteric stage: early menopause (≤4 years since last menstrual period, *n* = 126), intermediate postmenopause (5–8 years, *n* = 123), and advanced postmenopause (>9 years, *n* = 111). Cognitive performance was assessed using the Addenbrooke’s Cognitive Examination-Revised (ACE-R) and the Symbol Digit Modalities Test (SDMT). A Bayesian multivariate model with two-way interaction terms was fitted, simultaneously evaluating ACE-R and SDMT as response variables.

**Results:**

More advanced climacteric stage was associated with lower ACE-R and SDMT scores. Although the main effect of age at assessment showed high uncertainty, a significant age × climacteric stage interaction revealed that the age–cognition slope differed by stage. Specifically, in early menopause, older age was associated with better cognitive performance, whereas in advanced postmenopause, older age was associated with lower cognitive performance.

**Conclusion:**

The association between chronological age and cognitive performance differed according to climacteric stage in Chilean women. These findings challenge the traditional view of age as a uniformly negative correlate of cognition and may have implications for cognitive screening strategies during the menopausal transition.

## 1 Introduction

Women residing in extreme southern latitudes, such as Chile’s Magallanes Region (53°S), face a unique convergence of environmental and biological challenges during aging. The climacteric, the transition from reproductive to non-reproductive life, is characterized by profound neuroendocrine reorganization affecting multiple physiological systems, including brain health and cognitive performance (El Khoudary et al., 2019; Rocca et al., 2014). With global population aging, an estimated 1.2 billion women will be postmenopausal by 2030 (Faubion et al., 2015; Santoro et al., 2021). This demographic shift poses substantial challenges for healthcare systems in preventing cognitive decline during and after the menopausal transition. The menopausal transition has been consistently associated with changes in specific cognitive domains, particularly episodic memory, sustained attention, processing speed, and executive functions during the perimenopausal and early postmenopausal periods (Weber et al., 2013; Maki, 2013). These changes do not merely represent consequences of chronological aging but rather reflect a specific neurobiological reorganization driven by the hypoestrogenism characteristic of this stage (Than et al., 2023; Weber et al., 2014). The neurobiological mechanisms underlying this heterogeneity are multifactorial. Estrogens, particularly 17β-estradiol, exert neuroprotective effects through modulation of synaptic plasticity, cholinergic and dopaminergic neurotransmission, and attenuation of neuroinflammatory processes (Hampson, 2018; Kang et al., 2023; Baumgartner et al., 1998; Schelbaum et al., 2021). Progressive loss of these estrogenic neuroprotective effects may gradually compromise cognitive reserve mechanisms (Coughlan et al., 2023; Ramli et al., 2023), suggesting that the interaction between duration of hypoestrogenic exposure and chronological age could generate distinct neurocognitive trajectories according to climacteric stage. The relationship between chronological age and cognitive performance has traditionally been conceptualized as a progressive linear decline (Mensegere et al., 2024; Murman, 2015). This perspective has recently been questioned by evidence that the cognitive trajectory during female aging is modulated by endocrine, metabolic, and inflammatory factors that generate substantial individual heterogeneity (Salthouse, 2019; Kuh et al., 2018). Large-scale longitudinal and population-based studies have documented divergent cognitive profiles according to menopausal status (Georgakis et al., 2016; Greendale et al., 2009), while recent systematic reviews show considerable interindividual variability in perimenopausal cognitive decline (Fu et al., 2025; Brinton et al., 2015). Most methodological approaches have treated time since menopause and chronological age as independent variables or as reciprocal confounders requiring statistical adjustment (Henderson and Popat, 2011; Conde et al., 2021), assuming their effects on cognitive performance operate additively. While this approach has generated valuable knowledge about how each factor contributes to the neurocognitive profile, it leaves unaddressed a fundamental question: whether the relationship between age and cognition itself shifts according to menopausal transition phase. The existing literature recognizes that time since menopause and type of climacteric transition modulate cognitive decline risk (Gilsanz et al., 2019; Han et al., 2023), and recent meta-analyses identify the need to investigate how temporal variables and hormonal states interact (Prince et al., 2012). However, the possibility that chronological age may show different associations with cognitive performance across climacteric stages remains largely unexplored. Women of similar chronological age but at different climacteric stages may not only differ in cognitive performance but may also show opposite age–cognition associations across stages. Women in Chile’s Magallanes Region are exposed to unique environmental conditions, including extreme photoperiodic variation, harsh climate, and geographic isolation. These contextual characteristics may represent potential influences on health and aging processes, although their specific relationship with cognitive performance during menopause remains insufficiently characterized. Previous research in Latin American and South American populations has documented variability in cognitive and functional outcomes across diverse sociocultural and health contexts (Lebrão and Laurenti, 2005; Vallejo et al., 2025); however, evidence directly addressing the influence of environmental conditions such as those present in the Magallanes Region remains limited. The region’s geographic isolation and demographic composition create a context where the interaction between biological aging and climacteric stage may manifest differently than in more extensively studied populations. The present investigation addresses this gap through a methodological approach centered on interaction effects, examining whether the association between chronological age and cognitive performance differs according to climacteric stage in women from the Magallanes Region. The central hypothesis posits that the directionality and magnitude of the age-cognition association vary systematically across the climacteric transition. This hypothesis was tested using a Bayesian multivariate model with two-way interaction terms, simultaneously evaluating performance on the Addenbrooke’s Cognitive Examination-Revised (ACE-R) and the Symbol Digit Modalities Test (SDMT). This approach allows quantification of not only the independent effects of age and climacteric stage but their combined influence on cognition, specifically, whether the age–cognition association varies across different climacteric stages. To our knowledge, no previous studies have formally examined this modulation between chronological age and climacteric stage in cognitive performance.

## 2 Materials and methods

### 2.1 Study design

An observational, analytical, cross-sectional study was conducted. The evaluation protocol was submitted to ethical review and approved by the Ethics and Scientific Committee of Universidad José Camilo Cela (Resolution 21_25_NEUROCLIM). All procedures were carried out in adherence to the principles of the Declaration of Helsinki, and all participants signed informed consent before any evaluation.

### 2.2 Participants

Three hundred sixty women aged 50–81 years were recruited through convenience sampling across primary care centers, community organizations, and recreational facilities between January and November 2024. Participants were classified into three climacteric stages following STRAW+10 criteria (Harlow et al., 2012): early menopause (≤4 years since final menstrual period, *n* = 126), intermediate postmenopause (5–8 years since final menstrual period, *n* = 123), and advanced postmenopause (>9 years since final menstrual period, *n* = 111). This classification aligns with documented periods of accelerated hormonal change during early postmenopause and the stabilization phase in late postmenopause (Weber et al., 2014; Henderson, 2014). Inclusion criteria required: (a) age 50–81 years, (b) provision of written informed consent, and (c) complete menstrual history data allowing climacteric staging according to STRAW+10 criteria (Harlow et al., 2012). Exclusion criteria eliminated participants with: (a) neurological diseases (stroke, traumatic brain injury, epilepsy, Parkinson’s disease, dementia), (b) uncontrolled psychiatric conditions (major depression, bipolar disorder, schizophrenia, active substance dependence), (c) severe sensory deficits preventing cognitive evaluation, (d) current hormone replacement therapy, or (e) premature menopause (before age 40) or surgical menopause (bilateral oophorectomy).

### 2.3 Cognitive assessment

Global cognition was assessed using the Addenbrooke’s Cognitive Examination-Revised (ACE-R), a 100-point instrument evaluating attention/orientation (18 points), memory (26 points), fluency (14 points), language (26 points), and visuospatial abilities (16 points). The ACE-R demonstrates robust psychometric properties in Spanish-speaking populations and detects mild cognitive impairment with high sensitivity and specificity (Cancino et al., 2020).

Processing speed was measured with the Symbol Digit Modalities Test (SDMT), which requires participants to match symbols to numbers within 90 s. The SDMT exhibits strong test–retest reliability and convergent validity with other processing speed measures, with normative data available for diverse populations (Ryan et al., 2020).

Both instruments were administered by trained research assistants blinded to climacteric stage in standardized individual sessions lasting 45–60 min. All evaluations occurred in quiet, well-lit spaces during morning hours to minimize fatigue effects.

### 2.4 Sociodemographic and clinical data

Sociodemographic information including age, educational attainment (years of formal education), marital status, and employment status was collected through structured interviews. Clinical history documented age at menarche, parity, use of hormonal contraceptives, smoking status, alcohol consumption, physical activity level, and comorbidities (hypertension, diabetes, dyslipidemia, cardiovascular disease).

### 2.5 Statistical analysis

A Bayesian framework was employed to characterize changes in our variables of interest. Bayesian inference was preferred over traditional frequentist methods as it provides complete posterior distributions for all parameters, thereby allowing for a full quantification of uncertainty and a probabilistic interpretation via credible intervals. Descriptive statistics were reported as mean and standard deviation (M ± SD) for continuous variables and as absolute and relative frequencies [*n* (%)] for categorical variables. To assess the effect of climacteric stage and covariates on cognitive performance (SDMT and ACE-R scores), we fitted a multivariate Bayesian linear model. Climacteric stage was coded as an ordered categorical variable (early menopause < intermediate postmenopause < advanced postmenopause) using polynomial contrasts to capture linear and quadratic trends across stages. The model specified climacteric stage and age at menopause as primary predictors, with chronological age and educational level as covariates. Two-way interaction terms between climacteric stage and each covariate were included to assess modulation effects. This approach enabled direct comparison of effects across cognitive measures while accounting for their correlation. Additionally, a simpler model, without educational level as a covariate, was also fitted to show the aggregated effect of education over cognitive performance. Weakly informative priors, centered on a null effect, were used to exert a regularizing effect, reducing the influence of outliers and stochastic noise. All models were fitted using the No-U- Turn Sampler (NUTS) algorithm, a variant of Hamiltonian Monte Carlo (HMC). We ran 4 chains, with 10,000 warm-up iterations and 10,000 effective iterations per chain. Model convergence was confirmed by ensuring an R-hat statistic < 1.01 and an effective sample size (ESS) > 2,000, and by visually inspecting trace plots to confirm convergence to a stationary distribution.

Results were reported as standardized beta coefficients (*β*) with 95% credible intervals (CI95%) as the measure of effect size, along with the probability of direction (pd) and the probability of significance (ps). The pd. is the proportion of the posterior distribution that has the same sign as the median estimate. It ranges from 0.5 to 1.0. A value of 0.5 indicates that the posterior is perfectly symmetric around zero, meaning there is no more evidence for an effect than would be expected by chance. A value of 1.0 indicates that the entire posterior lies on one side of zero, implying complete certainty about the direction of the effect. Similarly, ps is the proportion of the posterior distribution that falls outside the region of practical equivalence, defined here as the interval from −0.1 to 0.1 standardized units. It can be interpreted as a measure of the probability that an effect is practically meaningful for standardized model estimates. All analyses used R 4.3.1 with brms 2.20.4 for Bayesian modeling and bayestestR 0.13.1 for evidence quantification. Given the inherent relationship between chronological age and climacteric stage, age was additionally centered to reduce potential collinearity in interaction terms. Sensitivity analyses using alternative model specifications yielded consistent results, supporting the robustness of the findings. Although additional clinical and lifestyle variables (e.g., comorbidities, smoking, alcohol use, physical activity) were collected, they were not included in the primary model in order to preserve parsimony and avoid overparameterization, given the inclusion of multiple interaction terms. These variables were considered as potential confounders and are addressed in the interpretation of the findings.

## 3 Results

### 3.1 Sample characteristics

The total sample collected consisted of 360 females with mean age 63.5 ± 8.0. Regarding climacteric stage, 126 (35.0%) were on early stage, 123 (34.2%) on intermediate stage and 111 (30.8%) on advanced climacteric stage. As expected, substantial differences were observed across groups in chronological age and time since menopause, reflecting the inherent structure of climacteric staging. These variables may independently influence cognitive performance and should be considered when interpreting group differences. Further sample characteristics can be observed in Table 1.

**Table 1:** Sociodemographic characteristics of the overall collected sample and aggregated by climacteric stage.

Characteristic	Overall *N* = 360^a^	Early stage *N* = 126^a^	Intermediate stage *N* = 123^a^	Advanced stage *N* = 111^a^
Age (years old)	63.5 ± 8.0	60.5 ± 6.8	60.9 ± 7.4	69.9 ± 6.2
Educational level				
Primary education	117 (33%)	35 (28%)	40 (33%)	42 (38%)
Secondary education	126 (35%)	46 (37%)	41 (33%)	39 (35%)
Higher education	117 (33%)	45 (36%)	42 (34%)	30 (27%)
Age at menopause diagnostic (years old)	54.6 ± 7.3	58.6 ± 7.0	54.9 ± 7.6	49.5 ± 3.2
Time after menopause diagnostic (years)	9.0 ± 8.7	1.8 ± 0.9	6.0 ± 1.4	20.4 ± 6.4
ACE-R | Total score	86.9 ± 2.9	88.7 ± 1.0	88.5 ± 0.7	83.2 ± 2.2
ACE-R | Orientation score	17.1 ± 0.8	17.0 ± 0.7	16.5 ± 0.5	17.8 ± 0.4
ACE-R | Memory score	18.4 ± 4.8	20.9 ± 1.1	21.6 ± 1.1	12.1 ± 3.8
ACE-R | Fluencies score	12.5 ± 1.0	11.9 ± 0.7	12.0 ± 0.6	13.6 ± 0.7
ACE-R | Language score	24.5 ± 0.9	24.3 ± 0.6	24.0 ± 0.6	25.4 ± 0.9
ACE-R | Visoespacial score	14.4 ± 0.7	14.6 ± 0.6	14.4 ± 0.7	14.3 ± 0.6
SDMT | Errors (N°)	1.3 ± 1.3	1.1 ± 1.2	1.0 ± 1.2	1.9 ± 1.3
SDMT | Velocity (%)	50.8 ± 6.9	52.7 ± 6.1	52.4 ± 6.2	46.9 ± 7.0
SDMT | Precision (%)	97.3 ± 2.8	97.8 ± 2.4	98.0 ± 2.6	96.0 ± 3.0
SDMT | Combined efficiency	32.0 ± 20.1	34.9 ± 20.2	37.3 ± 19.9	22.9 ± 17.3
SDMT | Total score	49.6 ± 7.7	51.7 ± 6.7	51.5 ± 7.0	45.1 ± 7.6

Descriptive statistics are presented as mean and standard deviation for continuous variables, whereas for categorical variables the data is presented as absolute (n) and relative (%) frequencies. ACE-R, Addenbrook Cognitive Examination Revised; SDMT, Symbol Digit Modalities Test.

a Values are presented as mean ± standard deviation for continuous variables and as absolute (n) and relative (%) frequencies for categorical variables.

### 3.2 Main effects of climacteric stage and age

When assessing the effect of climacteric stage on cognitive scores, we observed a marked linear negative effect with SDMT scores [𝛽 = − 1.09, CI95% (−1.8, −0.36), pd. = 99.9%, ps = 99.7%] and ACE-R total scores [𝛽 = − 1.49, CI95% (−1.88, −1.11), pd. = 100%, ps = 100%], suggesting that being in a late stage of menopause is associated with lower cognitive functioning levels. Similar findings, but with less than 80% posterior probability, were founded for age and its effect on SDMT [𝛽 = − 0.18, CI95% (−0.63, 0.26), pd. = 79.3%, ps = 64.4%] and ACE-R scores, whereas in this last one, the direction of effect was positive [𝛽 = 0.09, CI95% (−0.15, 0.32), pd. = 76.7%, ps = 46.9%]. These posterior probabilities indicate high certainty regarding the negative association between climacteric stage and cognitive performance, whereas the effect of age alone showed substantial uncertainty.

### 3.3 Critical interaction: chronological age × climacteric stage

However, when inspecting the interaction terms (i.e., how the effect changes when accounting for different covariates combinations), we observed that the effect of climacteric stage was dependent on age, This means that, among women in the early climacteric stage, older age was associated with better cognitive performance, for both SDMT scores [interaction effect, 𝛽= − 1.52, CI95% (−2.32, −0.72), pd. = 100%, ps = 100%] and ACE-R scores [interaction effect, 𝛽= − 1.4, CI95% (−1.83, 0.97), pd. = 100%, ps = 100%], whereas in advanced climacteric stages, older age was associated with lower cognitive performance. No strong evidence was found for an association between educational level and cognitive performance in this sample [SDMT, 𝛽 = 0.07, CI95% (0.06, 0.2), pd. = 83.9%, ps = 31%; ACE-R, 𝛽=0.02, CI95% (−0.09, 0.05), pd. = 73.4%, ps = 1.4%], after adjusting for confounding factors.

**Figure 1:**
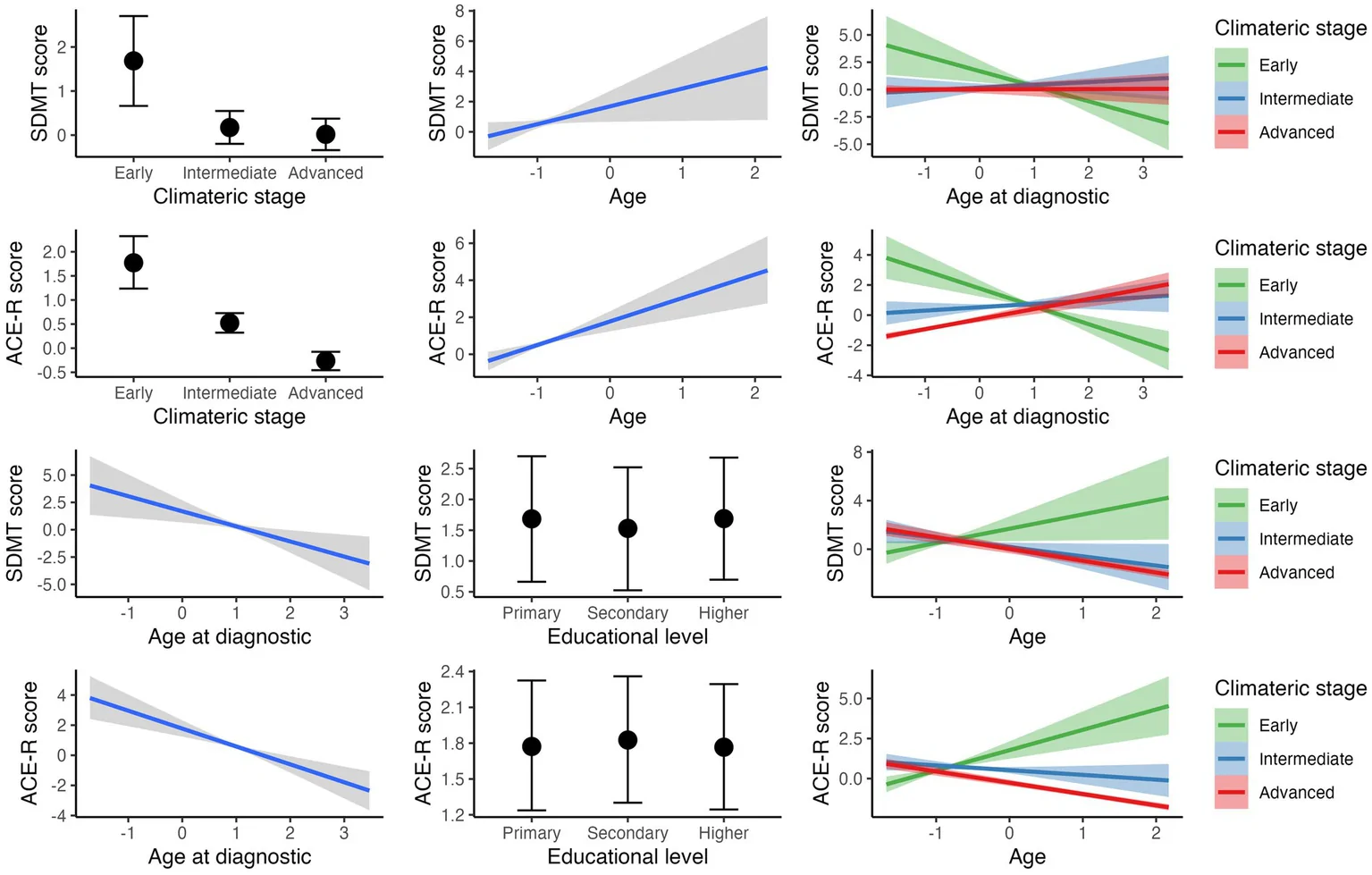
Conditional effects from the Bayesian multivariate model illustrating the interaction between chronological age and climacteric stage on cognitive performance. Lines represent posterior mean estimates, and shaded areas represent 95% credible intervals. The figure highlights stage-dependent differences in the association between age and cognitive outcomes (ACE-R and SDMT). Solid lines represent posterior mean estimates and shaded areas represent 95% credible intervals. The *x*-axis represents chronological age (years), and the *y*-axis represents predicted cognitive performance in standardized units. Positive slopes indicate that older age was associated with better cognitive performance within a stage, whereas negative slopes indicate an association with lower performance.

Conditional effects from multivariate Bayesian model are depicted in Figure 1.

## 4 Discussion

This investigation examined whether chronological age differentially modulates cognitive performance according to climacteric stage in Chilean women from the Magallanes Region. An important methodological consideration is the structural dependency between chronological age and climacteric stage, as women in more advanced stages are necessarily older. This relationship introduces potential collinearity and may partially influence the observed interaction effects. Although our modeling strategy incorporated both chronological age and age at menopause to account for shared variance, the possibility of residual confounding cannot be fully excluded. Therefore, the observed interaction should be interpreted with caution as a stage-dependent association rather than a fully independent effect The central finding was a stage-dependent age–cognition association across the reproductive transition: older age was associated with better cognitive performance in early menopause but with lower cognitive performance in advanced postmenopause. This interaction pattern was robust across two distinct cognitive measures, global cognition (i.e., ACE-R) and processing speed (i.e., SDMT), suggesting the effect generalizes beyond domain-specific vulnerabilities. The positive association between age and cognitive performance in early menopause challenges conventional expectations. Women entering postmenopause at older ages demonstrated better cognitive performance than those entering earlier, even after adjusting for education. This pattern may reflect several converging mechanisms. First, later age at menopause entry indicates prolonged estrogen exposure across the lifespan, which may have strengthened neural scaffolding and synaptic density in brain regions vulnerable to hypoestrogenic decline (Mosconi et al., 2021; Rahman et al., 2019). Second, women experiencing later menopause may have accumulated greater cognitive reserve through extended occupational engagement and social participation during their reproductive years (Stern et al., 2019). Third, the timing of menopause onset relative to chronological age could interact with developmental trajectories of brain aging, later transition may allow more time for compensatory neural mechanisms to consolidate before facing hypoestrogenic stress (Kantarci et al., 2018).

The reversal of this association in advanced postmenopause may suggest a potentially relevant temporal window. As duration of hypoestrogenic exposure increases beyond 8–9 years, the conventional age-cognition association reemerges. This shift may represent exhaustion of compensatory mechanisms that initially buffered against estrogen loss. The scaffolding theory of aging and cognition proposes that the brain recruits additional neural resources to maintain function as primary networks deteriorate (Reuter-Lorenz and Park, 2014). In early postmenopause, women with greater chronological age may possess more developed compensatory scaffolds. However, prolonged hypoestrogenism may eventually overwhelm these reserves, exposing underlying age-related vulnerabilities.

These findings diverge from previous research treating age and time since menopause as independent correlates or predictors of cognitive performance (Henderson and Popat, 2011; Conde et al., 2021). The interaction framework reveals that these temporal dimensions do not operate additively but instead reconfigure each other’s influence. A 60-year-old woman with 3 years since menopause exhibits a fundamentally different cognitive profile than a 60-year-old woman with 12 years since menopause, not merely because of accumulated time in hypoestrogenism, but because age itself functions differently in these contexts. This distinction carries profound implications for understanding individual trajectories through the menopausal transition.

The consistency of interaction effects across ACE-R and SDMT carries theoretical significance. The ACE-R captures multiple cognitive domains through complex tasks requiring memory encoding, semantic retrieval, and visuospatial processing. The SDMT measures processing speed through rapid symbol-digit matching. That both measures showed parallel interaction patterns suggests the age effect inversion operates at a level of neurocognitive organization that transcends specific cognitive domains. This convergence points toward systemic neurobiological mechanisms rather than isolated vulnerabilities in particular neural circuits.

The lack of a strong association between educational level and cognitive performance in this study should be interpreted with caution, given the extensive evidence supporting education as a key determinant of cognitive functioning. This finding may reflect limited variability in educational attainment within the sample, model specification, or the regularizing effect of Bayesian estimation. Several limitations warrant consideration. The cross-sectional design prevents causal inference or individual trajectory modeling. Longitudinal studies tracking women from perimenopause through late postmenopause could clarify whether the observed interaction represents genuine within-person change or reflects cohort effects. Hormonal biomarkers (estradiol, FSH) were not collected, limiting our ability to link cognitive patterns to specific endocrine profiles. Future research incorporating hormonal assessment could elucidate whether age effects are mediated by differential estrogen levels within climacteric stages. Additionally, relevant clinical and lifestyle factors such as comorbidities, smoking, alcohol use, and physical activity were not included in the multivariate model, which may introduce residual confounding in the observed associations. The sample derives from a specific geographic and cultural context, Chile’s Magallanes Region, limiting generalizability to other populations. However, this specificity also represents a strength, as most menopause research has focused on North American and European cohorts (Luetters et al., 2007). Moreover, comorbidities and lifestyle factors were not included into the models, which may have left potential residual confounding effects from these unmodelled factors. Future research should incorporate these moderating variables into account. The observed patterns carry direct implications for cognitive screening during menopause. Current clinical guidelines typically recommend age-stratified cognitive assessment without considering menopausal status. These findings suggest that chronological age thresholds for screening may need adjustment based on time since menopause. A 58-year-old woman 2 years postmenopausal may warrant less intensive monitoring than a 58-year-old woman 10 years postmenopausal, despite identical chronological age. Conversely, younger women in early postmenopause showing cognitive complaints should not be dismissed merely because of their age, their age may reflect a different cognitive profile within that climacteric stage rather than uniformly lower cognitive vulnerability. Importantly, no hormonal biomarkers were collected; therefore, the proposed biological mechanisms should be interpreted as hypothetical and not as directly demonstrated.

## 5 Conclusion

The association between chronological age and cognitive performance showed distinct patterns across climacteric stages in Chilean women. Older age was associated with better performance in early menopause but with lower performance in advanced postmenopause, consistently across global cognition and processing speed measures. These findings challenge linear interpretations of age related cognitive decline and emphasize the relevance of menopausal stage in the interpretation of cognitive outcomes. Future research should incorporate longitudinal designs and hormonal biomarkers to better understand the mechanisms underlying these differences.

## Data Availability

The raw data supporting the conclusions of this article will be made available by the authors, without undue reservation.

## Supplementary material

SUPPLEMENTARY FIGURE S1 Mean difference in model estimates from multivariate Bayesian linear model of SDMT **(A)** and ACE-R scores **(B)**, for the main terms and interaction effects, from the model using education as a covariate, compared to the model not using it. Distributions centered around the null standardized effect indicate that there is no difference in the model estimates between the model that uses education as a covariate from the model that does not use it. All estimates are adjusted for age and residual correlation between SDMT and ACE-R scores. All scores are on standardized units. ACE-R, Addenbrook Cognitive Examination Revised; SDMT, Symbol Digit Modalities Test; [L], linear effect from ordinal contrast; [Q], quadratic effect from ordinal contrast; Sigma, standard deviation of the normal distribution.

SUPPLEMENTARY FIGURE S2 Traceplots of main model estimates from multivariate Bayesian linear model of SDMT **(A)** and ACE-R scores **(B)**, illustrating model convergence to its stationary distribution. All estimates are adjusted for confounding factors and residual correlation between SDMT and ACE-R scores. All scores are on standardized units. ACE-R, Addenbrook Cognitive Examination Revised; SDMT, Symbol Digit Modalities Test; [L], linear effect from ordinal contrast; [Q], quadratic effect from ordinal contrast; Sigma, standard deviation of the normal distribution.
