# Correction: Chronological age is differentially associated with cognitive performance according to climacteric stage: evidence from a Bayesian multivariate analysis in Chilean women

**DOI:** 10.3389/fpsyg.2026.1997837

**Published:** 2026-10-07

**Authors:** Jonathan Lühr-Henríquez, Matías Castillo-Aguilar, Rosa Jurado-Barba, Cinthya Velásquez Muñoz, Cristian Núñez-Espinosa

**Affiliations:** 1 Escuela de Doctorado, Facultad HM de Ciencias de la Salud, Universidad José Camilo Cela, Madrid, Spain; 2 Departamento de Psicología, Universidad de Magallanes, Punta Arenas, Chile; 3 Escuela de Medicina, Universidad de Magallanes, Punta Arenas, Chile; 4 Faculty of Health Sciences - HM Hospitals, Camilo Jose Cela University, Madrid, Spain; 5 HM Hospitals Health Research Institute, Madrid, Spain

**Keywords:** ACE-R, age, Bayesian analysis, climacteric stage, cognition, menopause, SDMT

In the published article, affiliation “Faculty of Health Sciences - HM Hospitals, Camilo Jose Cela University, Madrid, Spain” and “HM Hospitals Health Research Institute, Madrid, Spain” were omitted from the paper. These affiliations have now been added for author Rosa Jurado-Barba.

The affiliation “Departamento de Psicología, Universidad de Magallanes, Punta Arenas, Chile” was erroneously included for author Rosa Jurado-Barba. This affiliation has now been removed for this author.

The original version of this article has been updated.

