# Supplementary figures and images for: Chronological age is differentially associated with cognitive performance according to climacteric stage: evidence from a Bayesian multivariate analysis in Chilean women
Source: Front Psychol. 2026 May 29;17:1823236. doi: 10.3389/fpsyg.2026.1823236 (PMC13259755; doi:10.3389/fpsyg.2026.1823236)

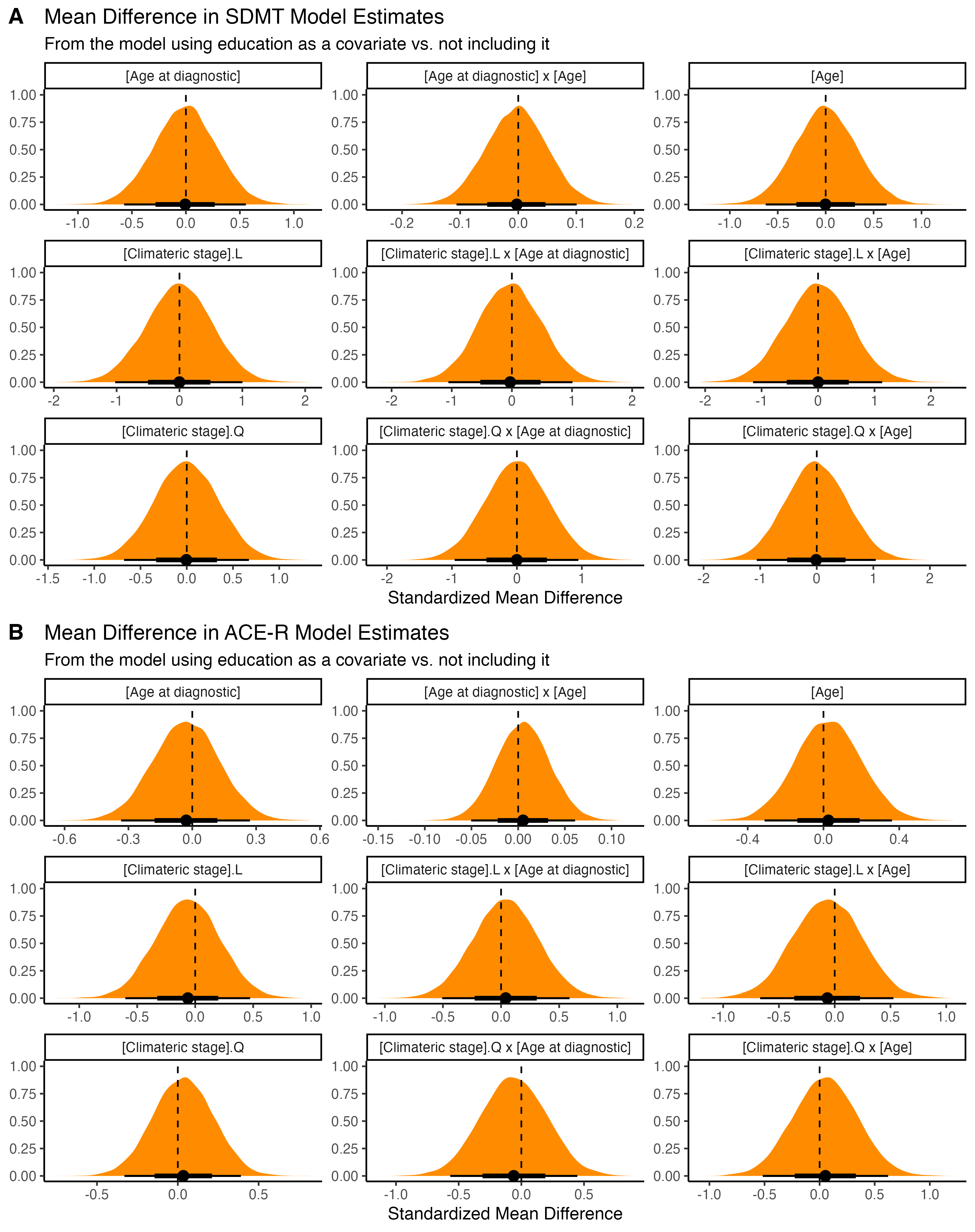

Supplement: SUPPLEMENTARY FIGURE S1 — Mean difference in model estimates from multivariate Bayesian linear model of SDMT (A) and ACE-R scores (B), for the main terms and interaction effects, from the model using education as a covariate, compared to the model not using it. Distributions centered around the null standardized effect indicate that there is no difference in the model estimates between the model that uses education as a covariate from the model that does not use it. All estimates are adjusted for age and residual correlation between SDMT and ACE-R scores. All scores are on standardized units. ACE-R, Addenbrook Cognitive Examination Revised; SDMT, Symbol Digit Modalities Test; [L], linear effect from ordinal contrast; [Q], quadratic effect from ordinal contrast; Sigma, standard deviation of the normal distribution. [file Image_1.PNG]

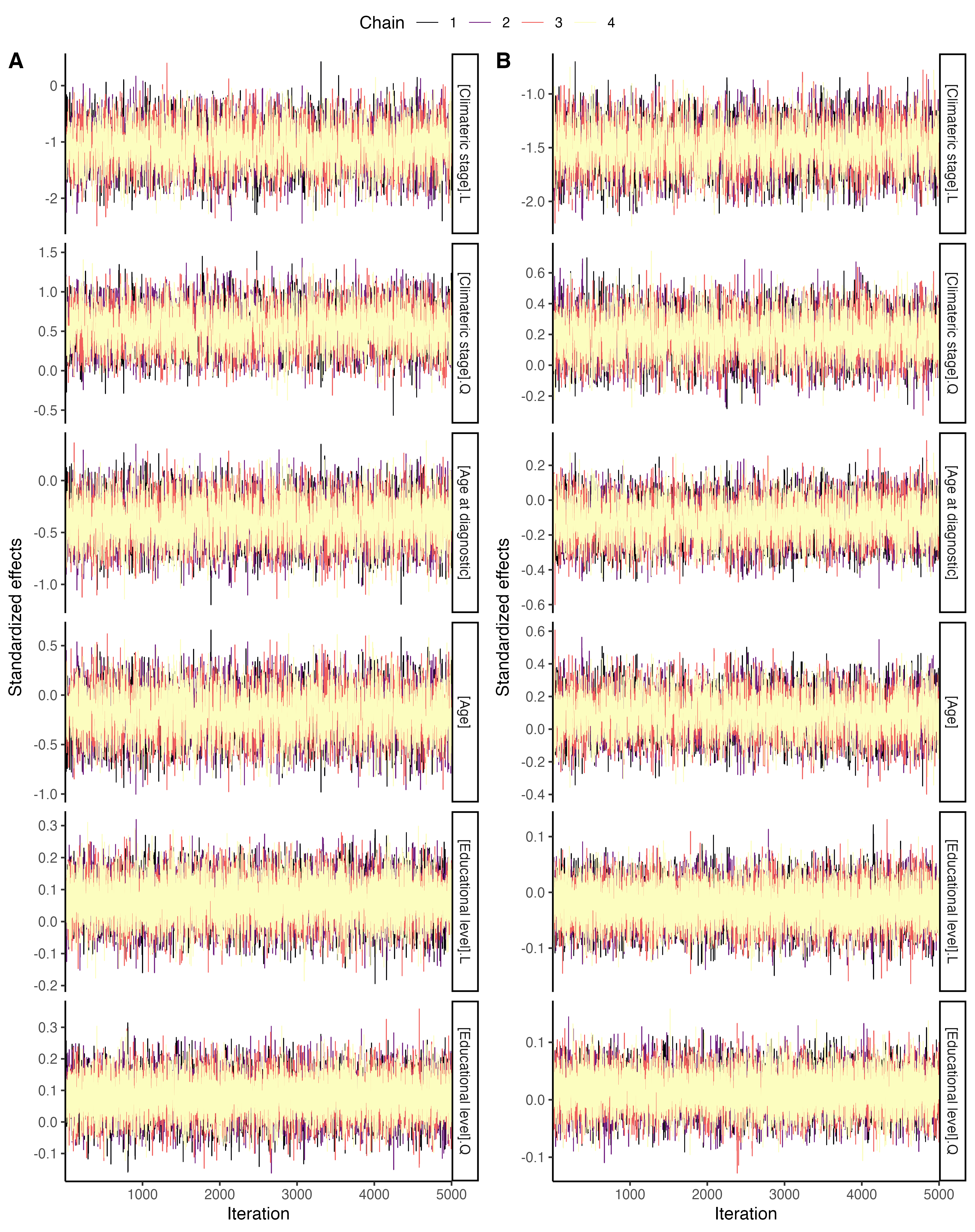

Supplement: SUPPLEMENTARY FIGURE S2 — Traceplots of main model estimates from multivariate Bayesian linear model of SDMT (A) and ACE-R scores (B), illustrating model convergence to its stationary distribution. All estimates are adjusted for confounding factors and residual correlation between SDMT and ACE-R scores. All scores are on standardized units. ACE-R, Addenbrook Cognitive Examination Revised; SDMT, Symbol Digit Modalities Test; [L], linear effect from ordinal contrast; [Q], quadratic effect from ordinal contrast; Sigma, standard deviation of the normal distribution. [file Image_2.PNG]
