# Supplementary material for: Chronological age is differentially associated with cognitive performance according to climacteric stage: evidence from a Bayesian multivariate analysis in Chilean women
Source: Front Psychol. 2026 May 29;17:1823236. doi: 10.3389/fpsyg.2026.1823236 (PMC13259755; doi:10.3389/fpsyg.2026.1823236)
Supplement: Supplementary file 3 [file Table_1.docx]

**Supplementary Table 1.** Posterior estimates from the Bayesian multivariate model: main effects and interaction terms for ACE-R and SDMT cognitive scores in Chilean women during the menopausal transition.

| **Response** | **Parameter** | **Estimate** | **95% CI** | **PD** | **PS** | **ESS** | **R-hat** |
| --- | --- | --- | --- | --- | --- | --- | --- |
| SDMT score | Intercept | 0.64 | [0.28, 1.01] | 1.000 | 0.998 | 5745.7 | 1.000 |
|  | Climateric stage [L] | -1.09 | [-1.8, -0.36] | 0.999 | 0.997 | 4950.3 | 1.001 |
|  | Climateric stage [Q] | 0.55 | [0.06, 1.02] | 0.987 | 0.964 | 5444.7 | 1.001 |
|  | Age at menopause diagnostic | -0.39 | [-0.79, 0.01] | 0.970 | 0.922 | 6780.0 | 1.000 |
|  | Age | -0.18 | [-0.63, 0.26] | 0.793 | 0.644 | 6439.2 | 1.000 |
|  | Educational level [L] | 0.07 | [-0.06, 0.2] | 0.839 | 0.310 | 27577.5 | 1.000 |
|  | Educational level [Q] | 0.08 | [-0.05, 0.2] | 0.898 | 0.383 | 29192.8 | 1.000 |
|  | Climateric stage [L] x Age at menopause diagnostic | 0.99 | [0.25, 1.71] | 0.996 | 0.992 | 5098.2 | 1.001 |
|  | Climateric stage [Q] x Age at menopause diagnostic | -0.77 | [-1.43, -0.07] | 0.986 | 0.973 | 6901.6 | 1.000 |
|  | Climateric stage [L] x Age | -1.52 | [-2.32, -0.72] | 1.000 | 1.000 | 4742.1 | 1.001 |
|  | Climateric stage [Q] x Age | 0.71 | [-0.06, 1.46] | 0.966 | 0.942 | 6786.0 | 1.000 |
|  | Age at menopause diagnostic x Age | -0.23 | [-0.3, -0.15] | 1.000 | 1.000 | 25886.4 | 1.000 |
|  | Climateric stage [L] x Educational level [L] | 0.13 | [-0.12, 0.39] | 0.844 | 0.599 | 23665.4 | 1.000 |
|  | Climateric stage [Q] x Educational level [L] | 0.07 | [-0.16, 0.28] | 0.723 | 0.378 | 28077.5 | 1.000 |
|  | Climateric stage [L] x Educational level [Q] | 0.01 | [-0.24, 0.27] | 0.523 | 0.238 | 20504.3 | 1.000 |
|  | Climateric stage [Q] x Educational level [Q] | 0.14 | [-0.08, 0.35] | 0.896 | 0.629 | 28021.5 | 1.000 |
|  | Age at menopause diagnostic x Educational level [L] | -0.01 | [-0.16, 0.14] | 0.534 | 0.108 | 21412.4 | 1.000 |
|  | Age at menopause diagnostic x Educational level [Q] | 0.04 | [-0.12, 0.18] | 0.679 | 0.203 | 20763.0 | 1.000 |
|  | Sigma SDMT score | 0.69 | [0.64, 0.74] | 1.000 | 1.000 | 27175.7 | 1.000 |
| ACE-R score | Intercept | 0.66 | [0.46, 0.85] | 1.000 | 1.000 | 6185.4 | 1.000 |
|  | Climateric stage [L] | -1.49 | [-1.88, -1.11] | 1.000 | 1.000 | 5301.0 | 1.000 |
|  | Climateric stage [Q] | 0.19 | [-0.07, 0.44] | 0.927 | 0.752 | 6026.9 | 1.000 |
|  | Age at menopause diagnostic | -0.12 | [-0.33, 0.1] | 0.855 | 0.559 | 7062.6 | 1.000 |
|  | Age | 0.09 | [-0.15, 0.32] | 0.767 | 0.469 | 6679.5 | 1.000 |
|  | Educational level [L] | -0.02 | [-0.09, 0.05] | 0.734 | 0.014 | 31731.7 | 1.000 |
|  | Educational level [Q] | 0.01 | [-0.05, 0.08] | 0.664 | 0.006 | 29490.1 | 1.000 |
|  | Climateric stage [L] x Age at menopause diagnostic | 1.31 | [0.93, 1.7] | 1.000 | 1.000 | 5519.8 | 1.000 |
|  | Climateric stage [Q] x Age at menopause diagnostic | -0.39 | [-0.77, -0.04] | 0.982 | 0.944 | 7582.8 | 1.000 |
|  | Climateric stage [L] x Age | -1.40 | [-1.83, -0.97] | 1.000 | 1.000 | 5113.2 | 1.000 |
|  | Climateric stage [Q] x Age | 0.48 | [0.06, 0.88] | 0.988 | 0.964 | 7428.7 | 1.000 |
|  | Age at menopause diagnostic x Age | -0.05 | [-0.09, -0.01] | 0.993 | 0.007 | 26111.6 | 1.000 |
|  | Climateric stage [L] x Educational level [L] | -0.02 | [-0.16, 0.11] | 0.635 | 0.132 | 23417.1 | 1.000 |
|  | Climateric stage [Q] x Educational level [L] | 0.00 | [-0.11, 0.12] | 0.517 | 0.050 | 30152.6 | 1.000 |
|  | Climateric stage [L] x Educational level [Q] | 0.09 | [-0.05, 0.23] | 0.890 | 0.423 | 20371.2 | 1.000 |
|  | Climateric stage [Q] x Educational level [Q] | 0.00 | [-0.12, 0.11] | 0.485 | 0.040 | 32834.6 | 1.000 |
|  | Age at menopause diagnostic x Educational level [L] | -0.05 | [-0.12, 0.03] | 0.881 | 0.092 | 20005.1 | 1.000 |
|  | Age at menopause diagnostic x Educational level [Q] | -0.04 | [-0.12, 0.04] | 0.824 | 0.062 | 19531.5 | 1.000 |
|  | Sigma ACE-R score | 0.37 | [0.34, 0.4] | 1.000 | 1.000 | 25441.9 | 1.000 |
| Both | Residual correlation: SDMT score & ACE-R score | 0.02 | [-0.09, 0.12] | 0.628 | 0.068 | 27754.4 | 1.000 |

Model estimates from multivariate Bayesian linear model from main terms and interaction effects. All effects are adjusted for confounders and residual correlation between SDMT and ACE-R scores. All scores are on standardized units. ACE-R, Addenbrook Cognitive Examination Revised; SDMT, Symbol Digit Modalities Test; CI, credible interval; PD, probability of direction; PS, probability of significance; ESS, effective sample size; R-hat, Gelman-Rubin statistic; [L], linear effect from ordinal contrast; [Q], quadratic effect from ordinal contrast; Sigma, standard deviation of the normal distribution.
